# Phenotypic Screening Approaches to Develop Aurora Kinase Inhibitors: Drug Discovery Perspectives

**DOI:** 10.3389/fonc.2015.00299

**Published:** 2016-01-07

**Authors:** Carlos Marugán, Raquel Torres, María José Lallena

**Affiliations:** ^1^Discovery Chemistry Research and Technology, Lilly Research Laboratories, Eli Lilly and Company, Alcobendas, Spain

**Keywords:** high-content imaging, assay development, cell cycle, pH3S10, Aurora kinases

## Abstract

Targeting mitotic regulators as a strategy to fight cancer implies the development of drugs against key proteins, such as Aurora-A and -B. Current drugs, which target mitosis through a general mechanism of action (stabilization/destabilization of microtubules), have several side effects (neutropenia, alopecia, and emesis). Pharmaceutical companies aim at avoiding these unwanted effects by generating improved and selective drugs that increase the quality of life of the patients. However, the development of these drugs is an ambitious task that involves testing thousands of compounds through biochemical and cell-based assays. In addition, molecules usually target complex biological processes, involving several proteins and different molecular pathways, further emphasizing the need for high-throughput screening techniques and multiplexing technologies in order to identify drugs with the desired phenotype. We will briefly describe two multiplexing technologies [high-content imaging (HCI) and flow cytometry] and two key processes for drug discovery research (assay development and validation) following our own published industry quality standards. We will further focus on HCI as a useful tool for phenotypic screening and will provide a concrete example of HCI assay to detect Aurora-A or -B selective inhibitors discriminating the off-target effects related to the inhibition of other cell cycle or non-cell cycle key regulators. Finally, we will describe other assays that can help to characterize the *in vitro* pharmacology of the inhibitors.

## Introduction

Aurora-A and Aurora-B are two serine threonine kinases that regulate cell cycle progression from G2 through to cytokinesis in a coordinated manner even though their localization and activation timing during the cell cycle varies. Aurora-A is required for mitotic entry, centrosome maturation and separation, and chromosome alignment (1), whereas Aurora-B is involved in chromosome condensation, segregation, and cytokinesis by regulating microtubule kinetochore associations (2). Inhibition of any of these two kinases will produce a different phenotype, while Aurora-A inhibition delays mitotic entry and progression and accumulates cells in G2/M phase (3, 4), Aurora-B inhibition prevents proper alignment of chromosomes to the spindle plate, inhibits cytokinesis, and results in the formation of multinucleated cells (5). However, the fact that human Aurora-A and -B share 71% identity in its carboxy-terminal catalytic domain is critical for evaluating the specificity of inhibitors (6).

Classic antimitotic drugs such as spindle poisons (e.g., taxanes and vinka alkaloids) prevent microtubule dynamics. Since microtubules, besides its mitotic role, are also necessary for multiple cellular functions during interphase, the use of these drugs is associated with side-effects such as neurotoxicity that can lead to irreversible neuropathologies (7). As opposed to previous molecules, inhibitors against specific therapeutical targets and focused on patients with specific characteristics (patient tailoring) need to be discovered. The pharmaceutical industry is evolving to fulfill this need, a tendency that can be observed in the development of CDK inhibitors. With approximately 14 molecules in clinical trials, the first generation molecules are often pan-CDK inhibitors (e.g., flavopiridol), whereas the more recent molecules tend to focus in specific CDKs (e.g., palbociclib and abemaciclib against CDK4/6) (8). The same concept applies for molecules against Aurora kinases, where most clinical trials have focused on Aurora-A selective agents (53%) as opposed to Pan-Aurora (32%) and Aurora-B-specific compounds (15%) (9).

In cancer treatment, there are mainly two approaches to inhibit a target: small molecules (e.g., gemcitabine against lung and pancreatic cancer) and large molecules (e.g., trastuzumab against ERBB2-overexpressing/amplified tumors). Large molecules include recombinant proteins and monoclonal antibodies and are often referred to as “biotech” drugs. (10). Whereas large molecules tend to be administered intravenously, small molecules usually allow easier administration (oral) but tend to be less selective. To discover a small molecule against a new target, pharmaceutical companies usually test thousands of compounds through biochemical assays, followed by a reduced number of compounds through cell-based assays and an even minor quantity through *in vivo* assays. Testing such an amount of compounds rapidly required the development of automation platforms and other technologies that allow the use of high-throughput screening (HTS) techniques. Usually, the molecular targets for cancer therapy are involved in complex biological processes and they interact with others from the same or even different molecular pathways. This adds a degree of difficulty to drug discovery in general and to assay development in particular. All of the above highlights the need for multiplexing technologies that allow for the evaluation of several readouts in the same experiment. Both, on-target and off-target effects will indicate the selectivity of the compounds, which ultimately, together with oral administration and safety profile, are the main desirable properties of a final drug candidate.

## Multiplexing Technologies

Singleplex technologies such as cell viability assays fall short in guaranteeing that the observed cellular effect upon compound treatment is due to inhibiting the target of interest. Off-target effects could create false positives and considering the challenge of selective compound properties, new technologies to monitor phenotypic changes associated with target inhibition are required. High-content imaging (HCI) and flow cytometry are two of the most commonly used techniques.

### High-Content Imaging

Also called high-content screening, HCI is a technique where a few hundred or a few thousand perturbagens (compounds, drugs, siRNAs, and cDNAs) are tested and scores of parameters are recorded from each individual cell using multiple imaging channels. The readouts can be kinetic and single endpoint using live and fixed cells, respectively (11).

The technology is based on obtaining one or several images of every sample, usually placed in wells of 96-well, 384-well, or even 1536-well microplates to achieve high throughput. For that purpose, two major types of detectors can be used: digital cameras and photomultiplier tubes (PMTs). The images can later be analyzed and managed by using specific software that usually comes with the instrument.

The assay type is an immunocytofluorescence assay and the selection of the proper antibody that recognizes the protein of interest is of importance. Usually, a secondary antibody is used to increase specificity and amplify the signal. These secondary antibodies are conjugated with fluorescent dyes that have a wide variety of absorption and emission wavelengths, allowing multiplexing while minimizing overlapping spectra (e.g., Alexa Fluor^®^).

There are basically three types of instruments according to the detection technology used: wide field imagers (often built around inverted research microscopes), confocal HCA imagers (confocal microscopes, preferred for live cell imaging and best used for imaging small intra-cellular structures, small cells, complex 3-D structures and samples with strong background fluorescence), and laser scanning cytometers (conceptually similar to a flatbed scanner with laser beams scanned across the entire surface of the plate and fluorescence detected with PMTs, good at detecting cells but not subcellular features or processes) (11).

### Flow Cytometry

This technique goes back to the invention of the first devices based on the Coulter principle to sort cell populations (12). Nowadays, fluorescence-based methods are used for the detection of biomarkers, cell counting, and sorting.

One of the key principles of flow cytometry is a process called hydrodynamic focusing. Basically, the fluidics system of the machine allows it to order the sample in solution that has been injected (where particles are randomly distributed in three-dimensional space) into a stream of single particles that can be interrogated by the detection system. Subsequently, each particle passes through one or more beams of light. Light scattering or fluorescence emission provides information about the particle’s properties. The laser and the arc lamp are the most commonly used light sources in modern flow cytometry (13).

With the possibility to analyze single cell events out of cellular aggregates or clusters, flow cytometry overcomes one of the main disadvantages of HCI. However, working with formats such as 384-well plates in flow cytometry is more complex than in HCI, requiring additional optimization to improve signal homogeneity and reading time.

A wide array of phenotypic changes can be chosen as readout: changes in morphology, protein translocation and expression, alteration of the phosphorylation status of proteins, changes in DNA content (e.g., Aur B inhibition usually leads to endoreplication and an increase of cells with 8N and beyond), and epigenetic modifications (e.g., H3K27). Both, HCI and flow cytometry allow for the development of novel cell-based assays to define the *in vitro* efficacy of new molecules. Through a combination of the multiple readouts mentioned above, we can detect both on-target and off-target effects of the drug and can evaluate the most appropriate concentration of a drug and the accurate cellular exposure to achieve the desired phenotype. These parameters are of importance for the design of the *in vivo* dosing schedule and eventually for a successful drug discovery process.

## Implementation, Optimization, and Validation of High-Throughput Assays

### Assay Development

**Figure 1:**
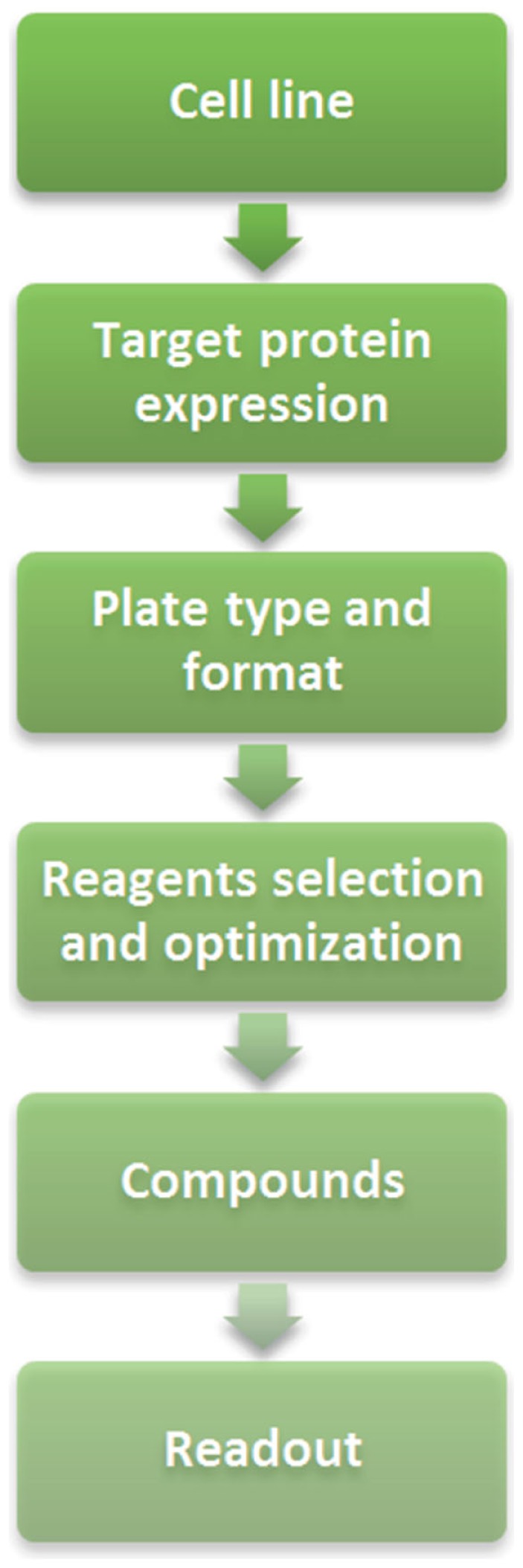
**Assay development flow chart**.

When setting up a cell-based assay for screening for the first time, there are several steps that should be followed [Figure 1; (11)]. One of the first steps is to select the cell lines for the study. Through the use of internal or external cell sensitivity panels and published scientific articles, cell lines with an anticipated response to the target of interest (e.g., Aurora kinases) could be identified. This will not only help to select the most appropriate cell lines for the assay but also open the possibility to study the genetic background of those cell lines to establish a connection with target inhibition leading to a proper patient-tailoring strategy. As a counterscreening for toxicity, a non-sensitive cell line can be selected or other readouts can be added to the sensitive cell lines (for example, apoptosis or senescence) to confirm the cause of cell proliferation inhibition.

Another parameter that would need to be considered is if and how the target protein is expressed in the cellular model of interest. This will provide an idea of the possible readouts for the assay: monitor directly changes in protein expression, phosphorylation, or location; surrogate readouts such as phenotypic changes; or even a combination of both.

Once the cell lines have been selected, according to the expected throughput of the assay generally either 96-well or 384-well plates will be chosen to seed the cells. Growth conditions and the appropriate cell seeding density will also need to be determined. Clear bottoms are required in these wells, so the lasers can excite the sample. If using poorly adherent cells, it is useful to plate the cells in wells coated with extracellular matrix components (e.g., poly-d-lysine, collagen, etc.).

Fixing and staining steps are quite similar to those of an immunofluorescence assay. The reagents used can be optimized according to the cell line and antibody that have been selected. Reagents to be optimized include salt-based solutions (Hank’s balanced salt solution, phosphate-buffered saline, and Tris-buffered saline), fixatives (formaldehyde, methanol, or other non-toxic fixative reagents), permeabilization buffers (salt solutions or water containing detergents, such as Triton X-100, Tween-20, SDS, and NP-40), blocking buffers (BSA, milk, and FBS), and the antibodies (concentration).

It is critical which type of compounds will be used (agonists, antagonists or both) and whether there is an available reference compound. Moreover, the DMSO tolerance of the cells and the period of time that the cells will be incubated with the compounds are important factors for the assay. The treatment duration depends on the type of response and the doubling time of the cell line used, e.g., changes in phosphorylation usually can be monitored within hours whereas changes in DNA content will require more time.

The number of lasers and detection channels available in the HCI instrument is essential for the readouts for the assay. These instruments will allow the use of DAPI/Hoechst or propidium iodide to stain the nuclei and different secondary antibodies.

#### Setting Up a Flow Scheme

Biochemical assays are an easy way to rapidly evaluate thousands of molecules and select a reduced number of molecules for their further characterization in cell-based assays.

Basically, several biochemical assays are set up for the enzymes of interest and others closely related, either from the same family (Aurora-A and -B) or involved in the same pathway (CDKs, PLK1, etc.). Usually, the inhibition of the latter ones should be avoided to ensure that the phenotypic outcome of the cell-based assay is due to inhibition of the target of interest (Aurora). Several techniques can be used to monitor biochemically the effect produced by the compounds: radioactivity, fluorescence, luminescence, mass spectrometry, etc. These assays will allow for the selection of the best molecules according to potency and selectivity to be tested in the cell-based assays.

Both, biochemical and cell-based assays, along with novel biophysical techniques, are used to evaluate structure–activity relationship (SAR) and design improved versions of the molecule. The more advanced molecules would be studied in-depth by testing several cell lines to confirm a link between genetic background and drug sensitivity. Drug combination with standards of care could also be addressed. Finally, the molecules will be tested *in vivo* to confirm the efficacy, and to evaluate whether they will proceed to clinical studies.

Thus, the flow scheme determines the different stages the compounds will go through before determining a candidate molecule for clinical trials. The assays included in the flow scheme need to be biologically significant for the targeted disease and the different stages need to show a desirable degree of connectivity.

### Assay Validation

Due to the high number of compounds to be evaluated, the assays need to be reproducible overtime and independently of the operator performing the assay. For that reason, there is a clear need for strict assay validation criteria that assure high quality data. When validating a new assay, we will require two different types of validation assays: a 3-day plate uniformity study and a replicate-experiment study.

#### Plate Uniformity

Uniformity assays are performed at the maximum and minimum signal or response levels to ensure that the signal window is adequate to detect active compounds during the screen. Therefore, the variability tests are conducted on three types of signals: “Max” signal (the maximum signal as determined by the assay design), “Min” signal (the background signal as determined by the assays design), and also “Mid” signal (this parameter estimates the signal variability at some point between the maximum and minimum signals).

**Figure 2:**
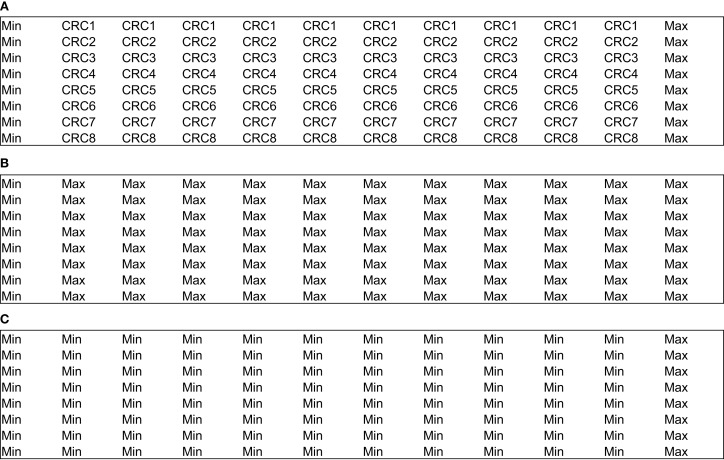
**Example of concentration–response curve plate format for plate uniformity assessment (96-well plates)**. **(A)** CRC plate. **(B)** “Max” plate. **(C)** “Min” plate.

Two different plate formats exist for the plate uniformity studies: interleaved-signal format – where all signals are on all plates but varied systematically, so that on all plates, on a given day, each signal is measured in each plate; and concentration–response curve plate format – where a reference compound is tested at multiple concentrations with production control wells (Max and Min, Figure 2A). The last one also includes uniform signal plates for “Max” (Figure 2B) and “Min” (Figure 2C) where each signal is run uniformly instead of the concentration–response curve for the reference compound. In both cases, the recommended acceptance criterion is *Z*′ factor ≥0.4 (which is comparable to a Signal Window ≥2), coefficient of variation <20%, absence of edge, drift or other spatial effects, and minimum significant ratio <3 or the normalized average Mid-signal should not translate into a twofold shift (within days or across any 2 days).

#### Replicate-Experiment Study

Replicate-experiment studies are used to formally evaluate the within-run assay variability and are a diagnostic and decision tool used to establish that the assay is ready to go into production by showing that the endpoints of the assay are reproducible over a range of potencies.

The analysis approach used in the replicate-experiment study is to estimate and factor out between-run variability, and then estimate the magnitude of within-run variability. The procedure has mainly three steps:

1. Select 20–30 compounds that have potencies covering the concentration range being tested and, if applicable, efficacy measures that cover the range of interest. The compounds should be well spaced over these ranges.
2. Run all compounds in each of two runs of the assay.
3. Compare the two runs. A series of statistical parameters will be calculated (mean-ratio, ratio limits, minimum significant ratio, and limits of agreement). MSR should be <3 and both limits of agreement should be between 0.33 and 3.0.

After successfully passing both validation studies (plate uniformity and replicate experiment), the assay is ready and the different libraries of compounds can be tested.

For a more comprehensive explanation on HTS assay validation, please refer to Iversen et al. (14).

## Practical Examples Applied to Aurora Inhibitors

### Development of pH3S10 and PI Multiplexing Assay

To evaluate Aurora-A or -B phenotype for different libraries of compounds, a multiplexing assay monitoring pH3S10 and DNA content was developed.

#### Cell Model

HeLa cells (ATCC# CCL-2) are epithelial cells isolated from cervix adenocarcinoma. These cells were selected based on their morphology for the imaging assay and because of the selected target of interest.

The optimal cell seeding density was evaluated and 5000 cells per well (96-well plates) was chosen as it produces a strong enough signal while cells remain well separated to allow single cell identification. To avoid loss of responsiveness, cells with as low passage number as possible were used and never exceeding a passage of 20. Cells were plated 18–24 h prior to compound dosing and were incubated at 37°C with 5% CO_2._

#### Compound Treatment

DMSO tolerance experiments determined that 0.25% DMSO should not be exceeded. As the cell doubling time is around 20 h, 24 h of incubation with compounds was chosen as an appropriate dosing time that would allow monitoring changes in mitotic index.

To perform compounds dose–response titration, threefold serial dilutions (in complete growth media containing 0.75% DMSO) were carried out in 96-well plates. This created a 10-point curve starting from 20 μM (final concentration in the assay). Then, 50 μL of compound solution was transferred from a dilution plate onto a cell plate containing 100 μL of culture media.

#### Assay Performance

HeLa cells were incubated with compounds for 24 h at 37°C/5% CO_2_, fixed with Prefer (Anatech) for 30 min at room temperature, and permeated with 0.1% Triton X-100 in PBS for 15 min. After a couple of washing steps with PBS, cells were blocked with 1% BSA in PBS. Then, the blocking solution was removed and the primary antibody solution [rabbit anti-phospho-histone H3 (ser10), Millipore] was added to the cells (1:1000 in 1% BSA in PBS) and they were incubated overnight at 4°C with a gentle shake. Next day, the primary antibody was washed away with PBS and cells were treated with 1:1000 Alexa Fluor 488-conjugated secondary antibody for 1 h at room temperature in the dark. Finally, upon washing steps with PBS, cells were treated with 1.4 μg/mL PI solution containing 50 μg/mL ribonuclease for 2 h at room temperature (DNA staining). An Acumen Explorer (TTP Labtech) was used for reading and image analysis.

**Figure 3:**
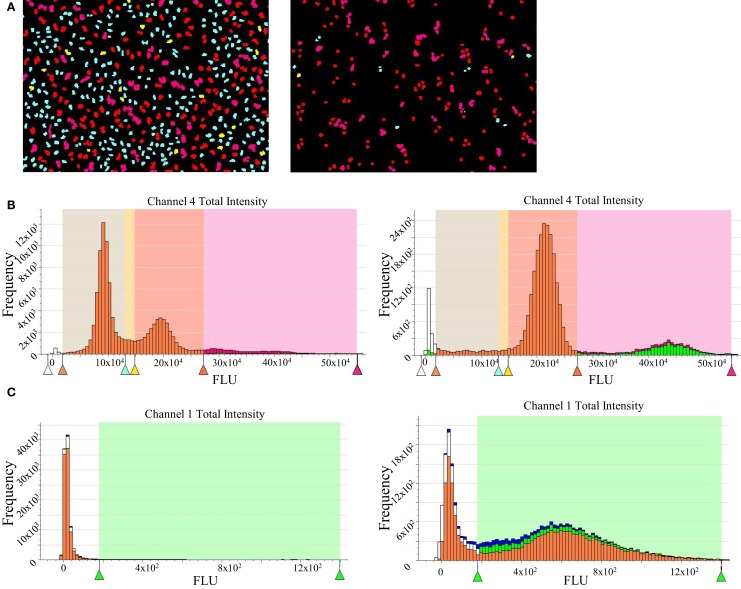
**High-content imaging data for propidium iodide staining and pH3 detection**. **(A)** Acumen screenshots of untreated (left) and nocodazole-treated HeLa cells (right). **(B)** Histograms showing cell cycle distribution generated after raw data processing from untreated (left) and nocodazole-treated cells (right). The G1 subpopulation is in cyan blue; S is represented in yellow, G2M phase in red, and cells with >4N DNA content are in pink. **(C)** Histogram showing pH3 signal after raw data processing from untreated (left) and nocodazole-treated cells (right).

Nocodazole was used as a tool compound for assay validation. It is a molecule that affects microtubule dynamics by preventing polymerization. This will result in the activation of the spindle assembly checkpoint and, therefore, a cell cycle arrest at G_2_/M phase. As a negative control, untreated cells were used. This assay allows the measurement of several readouts with single cell resolution and we focused our interest mainly in the following: cell number, 2N, 4N, >4N, and pH3S10. Aurora-B kinases are responsible for one of the classic modifications of chromatin in mitosis, phosphorylation of histone H3 on S10 (6), that is why pH3S10 was chosen as both mitotic marker and readout of Aurora-B inhibition. Data from treated and untreated cells are plotted to generate histograms that can be gated to separate diploid cells, tetraploid, and polyploid cells, and cells in S phase. As expected, nocodazole-treated cells show an accumulation in G_2_M as well as an accumulation of pH3 positive cells (Figure 3).

### Results Interpretation and Phenotype Deconvolution

**Figure 4:**
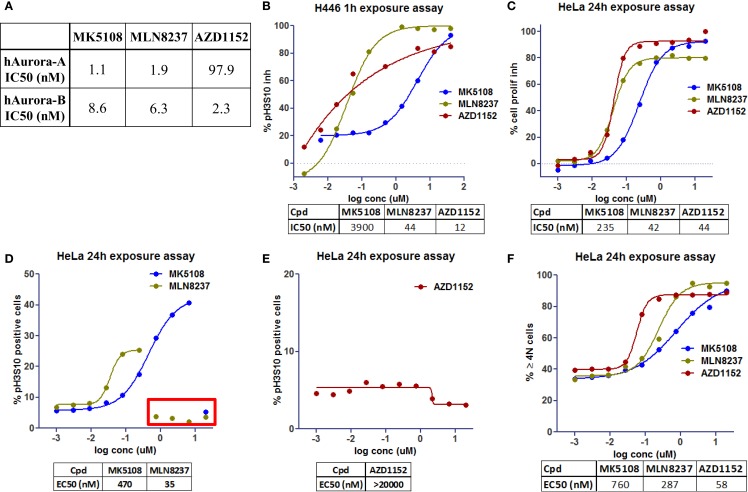
**Dose–response curves for Aurora kinases inhibitors in clinical trials [modified from Ref. (18)]**. **(A)** Inhibition of recombinant human Aurora-A and -B in biochemical assays. **(B)** Inhibition profile for pH3S10 after 1h of exposure (NCI-H446 cells). **(C)** Inhibition profile for cell proliferation after 24 h of exposure (HeLa cells). **(D,E)** Accumulation profile for pH3S10 after 24 h of exposure (HeLa cells). **(F)** Accumulation profile for cells with 4N and >4N DNA content after 24 h of exposure (HeLa cells).

To evaluate the phenotypic outcome of the assay and thus help with results’ interpretation, three molecules that are or have been in clinical trials showing different Aurora-A and -B selectivity profiles were selected: AZD1152, a selective Aurora-B inhibitor (15), and selective Aurora-A inhibitors, MLN8237 (16) and MK5108 (17). These molecules were first tested in biochemical assays (Figure 4A) to confirm their potency against Aurora-A and -B.

By performing 10-point dose–response curves in the previously mentioned multiplexing assay, we evaluated the effect of the compounds in the different readouts. Those readouts allow distinguishing Aurora-A and -B phenotypes, whereas Aurora-A leads to mitotic arrest (increase of 4N subpopulation and pH3S10, and therefore cell proliferation inhibition), Aurora-B leads to endoreplication (increase of 4N but mainly >4N subpopulations, cell proliferation inhibition, and a decrease in pH3S10).

To confirm the Aurora-B phenotype of the compounds a singleplex assay was also developed, using NCI-H446 cells (human small cell lung carcinoma, ATCC# HTB-171) and evaluating inhibition of pH3S10 at a shorter time (1 h incubation, Figure 4B).

AZD1152 shows a clear Aurora-B phenotype with pH3S10 inhibition and accumulation of >4N subpopulation (Figures 4B,E,F), whereas MLN8237 and MK5108 show an Aurora-A phenotype with accumulation of pH3S10 positive cells and 4N subpopulation (Figures 4D,F). With regards to cell proliferation inhibition (Figure 4C), MK5108 seems to be less potent than the other two molecules.

In Figure 4D, we can see inside the red rectangle, a possible effect not related to Aurora-A inhibition (higher in MLN than in MK) when evaluating pH3S10. At high concentrations of these compounds, there is a decrease in this readout that might be a consequence of Aurora-B inhibition (as seen in Figure 4B). Although in Figure 4F, the % of 4N and >4N positive cells was represented as one readout, it could be separated into two to further differentiate Aurora-A and -B phenotype.

By looking at the cell cycle subpopulations, with this type of assay we can also identify effects caused by inhibition of other targets (e.g., G_1_S arrest for CDK4/6 inhibitors).

### *In vitro* Pharmacological Characterization Through Multiplexing Assays

To further extend the use of this technology, more in-depth assays can be designed for advanced molecules as a bridging step between biochemical assays and *in vivo* assays. By using different exposure times to the compounds and performing washout experiments, we can try to investigate the *in vitro* pharmacology (required time on target and sustainability of the response). Adding different readouts as apoptosis or senescence will also help to identify the cause of cell proliferation inhibition.

**Figure 5:**
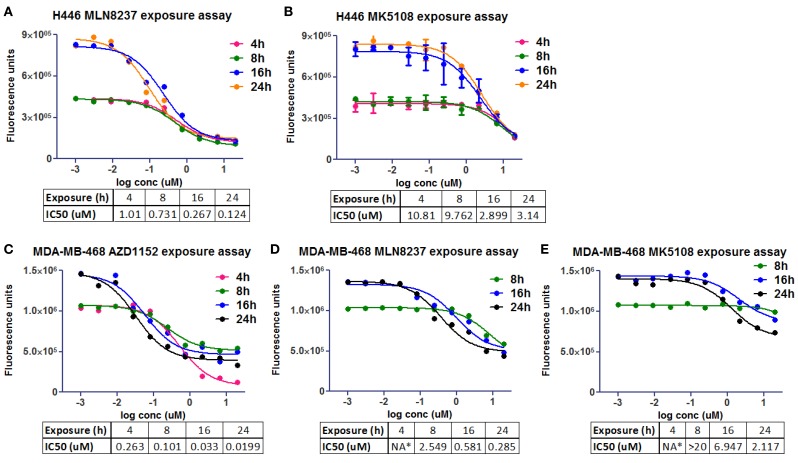
**Cell viability *in vitro* assays to determine the optimal pharmacological dose schedule for AZD1152, MLN8237, and MK5108 [modified from Ref. (18)]**. **(A,B)** H446 cells. **(C–E)** MDA-MB-468 cells. NA*, not available.

The same molecules were used in an experiment to estimate the exposure time needed in two different cell lines, NCI-H446 and MDA-MB-468 (human breast adenocarcinoma ATCC# HTB-132), to promote growth inhibition and achieve the desired phenotype (Figure 5). CellTiter-Glo^®^ was used to evaluate cell viability inhibition as one of the cell lines (NCI-H446) is mixed, with both adherent and suspension cells. It seemed that at least 24 h on target were required to promote cell growth inhibition.

**Figure 6:**
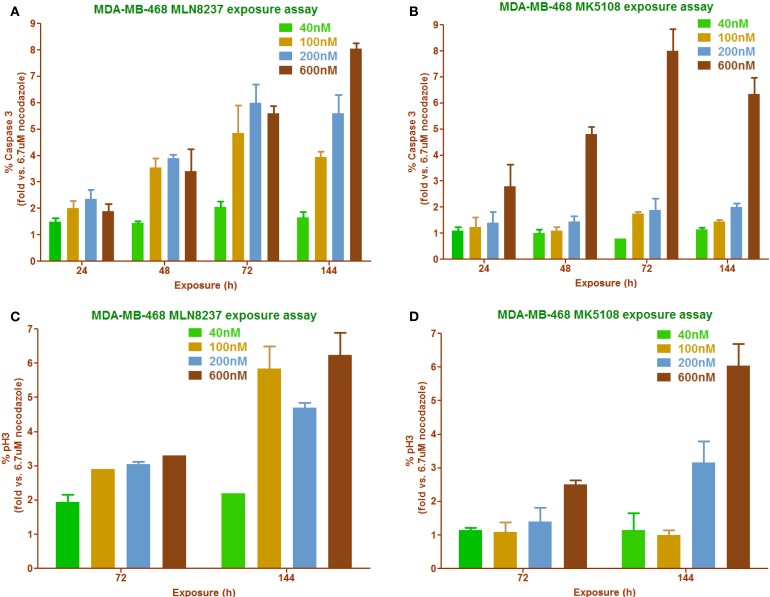
**Phenotypic readouts, permanent exposure to MLN8237 and MK5108** (18). **(A,B)** Percentage of caspase 3 positive cells determination in a time course manner carried out in a range of compounds concentrations. *n* = 2 per conc. **(C,D)** Percentage of mitotic index determination in a time course manner carried out in a range of compounds concentrations. *n* = 2 per conc.

High-content imaging follow-up experiments were performed in MDA-MB-468 to correlate cell proliferation inhibition with phenotypic readouts (% pH3S10 accumulation and % caspase 3 induction) as well as to try to find the most appropriate dose of the compounds to promote these effects (Figure 6). As already shown in Figure 4C, MK5108 was found to be less potent than MLN8237 when used at the same dose. This observation correlates with the two different readouts used in this experiment: caspase 3 (Figures 6A,B) and pH3S10 (Figures 6C,D), where MK5108 seems to require a higher dose (600 nM) and longer exposure time (72–144 h) to produce a considerable response, whereas MLN8237 seems to work at 200 nM and 48–72 h.

To summarize, we have reviewed a couple of multiplexing technologies focusing on HCI as a powerful technique for HTS. This technique can be used not only for screening purposes but also to go in-depth and try to characterize the *in vitro* pharmacology of the molecules. This could build a bridge between *in vitro* and *in vivo*, saving resources and helping to design more appropriate *in vivo* experiments. All of this integrated in a flow scheme will generate key data to select the best candidate molecule (with desired properties such as oral administration, safety, and selectivity) improving its possibilities to move into clinical trials.

## Conflict of Interest Statement

The authors declare that the research was conducted in the absence of any commercial or financial relationships that could be construed as a potential conflict of interest.
